# Bugs, genes, fatty acids, and serotonin: Unraveling inflammatory bowel disease?

**DOI:** 10.12688/f1000research.6456.1

**Published:** 2015-10-27

**Authors:** Jonathan Kaunitz, Piyush Nayyar

**Affiliations:** 1 Medical Service, West Los Angeles VAMC, Los Angeles, CA, 90073, USA; 2 Department of Medicine, David Geffen School of Medicine, Los Angeles, CA, 90095, USA; 3 Department of Surgery, David Geffen School of Medicine, Los Angeles, CA, 90095, USA

**Keywords:** Inflammatory bowel disease, ulcertative colitis, crohn's diseas, IBD

## Abstract

The annual incidence of the inflammatory bowel diseases (IBDs) ulcerative colitis and Crohn’s disease has increased at an alarming rate. Although the specific pathophysiology underlying IBD continues to be elusive, it is hypothesized that IBD results from an aberrant and persistent immune response directed against microbes or their products in the gut, facilitated by the genetic susceptibility of the host and intrinsic alterations in mucosal barrier function. In this review, we will describe advances in the understanding of how the interaction of host genetics and the intestinal microbiome contribute to the pathogenesis of IBD, with a focus on bacterial metabolites such as short chain fatty acids (SCFAs) as possible key signaling molecules.  In particular, we will describe alterations of the intestinal microbiota in IBD, focusing on how genetic loci affect the gut microbial phylogenetic distribution and the production of their major microbial metabolic product, SCFAs. We then describe how enteroendocrine cells and myenteric nerves express SCFA receptors that integrate networks such as the cholinergic and serotonergic neural systems and the glucagon-like peptide hormonal pathway, to modulate gut inflammation, permeability, and growth as part of an integrated model of IBD pathogenesis.  Through this integrative approach, we hope that novel hypotheses will emerge that will be tested in reductionist, hypothesis-driven studies in order to examine the interrelationship of these systems in the hope of better understanding IBD pathogenesis and to inform novel therapies.

## Introduction

Inflammatory bowel disease (IBD) is a term encompassing two major types of disorders—ulcerative colitis and Crohn’s disease—that are characterized by chronic relapsing intestinal inflammation ^1^. The incidence and prevalence of IBD has increased globally over the past few decades: in a systematic review of population-based IBD data, the average annual incidence rate was reported as 1.2–23.3% for Crohn’s disease and 2.4–18.1% for ulcerative colitis from 1920–2010 ^2^. Recent estimates of the total annual financial burden (including direct and indirect costs) of IBD in the US are $14.6–$31.6 billion ^3– 6^. Although newer therapies that have improved quality-of-life for a subset of patients have emerged in recent years, the underlying causes of and preventative measures against IBD remain unknown.

**Figure 1.**
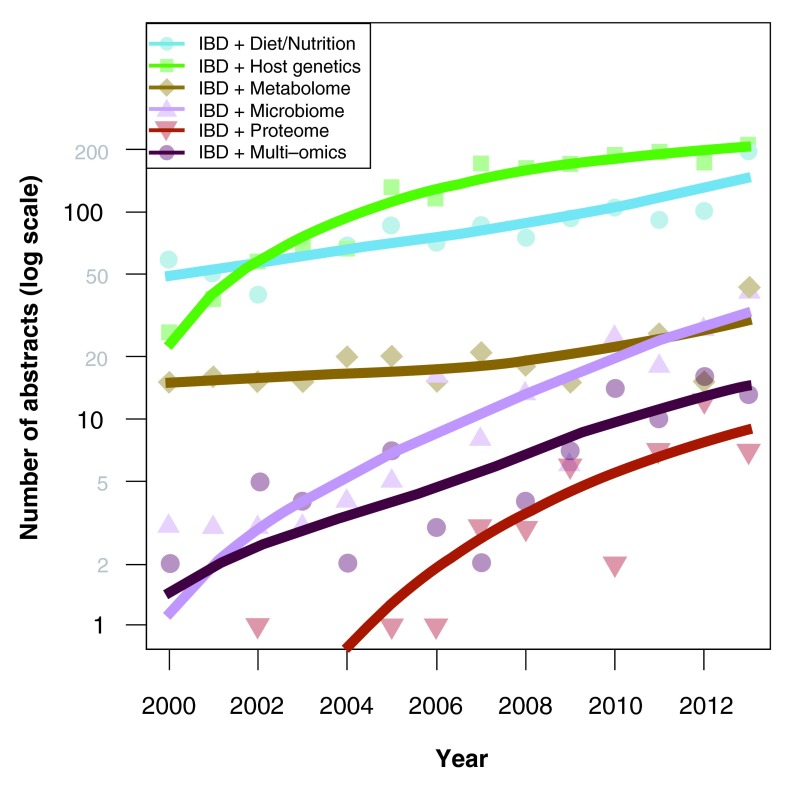
Historical trends of IBD research since the year 2000. The graph depicts increasing IBD-related research articles that focus on genetics and gut microbiome. Adapted from 7.

Major scientific advances over the last decade in the fields of genetics, immunology, and microbiology have increased our understanding of the underlying pathways involved in IBD. Although the specific pathophysiology continues to be elusive, it is hypothesized that IBD results from an aberrant and persistent immune response directed against microbes or their products in the gut, facilitated by the genetic susceptibility of the host and intrinsic alterations in mucosal barrier function. Figure 1 depicts the historical trends of increasing IBD-related research articles that focus on genetics and gut microbiome since the year 2000 ^7^.

We will describe advances in the understanding of how the interaction of host genetics and the intestinal microbiome contribute to the pathogenesis of IBD, with a focus on bacterial metabolites such as short chain fatty acids (SCFAs) as possible key signaling molecules.

## Genetics

**Figure 2.**
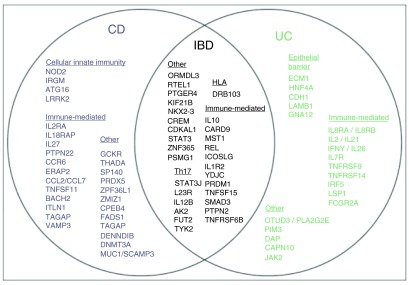
Genetic loci associated with IBD. IBD loci are represented by lead gene name and grouped by disease specificity and involved pathways. Loci associated with inflammatory bowel disease are shown in black, Crohn’s disease (CD) in blue, ulcerative colitis (UC) in green and both UC and CD in black. Adapted from 19.

The argument for a genetic predisposition to IBD begins with the observation that family members of affected persons have a greatly increased risk for developing IBD, with a relative risk 8–10 times higher amongst first-degree relatives of IBD patients compared with the general population ^8, 9^. Subsequent epidemiological data, which include differences in prevalence amongst different ethnic groups, familial aggregation of IBD, concordance in twins, and association with genetic syndromes, further confirmed the influence of genetics in IBD ^8– 13^. These instrumental early studies preceded the era of modern IBD genetic research with the discovery of the nucleotide-binding oligomerization domain containing 2 ( *NOD2*) gene in 2001, the first susceptibility gene discovered for Crohn’s disease ^14– 16^. In an analysis of 75,000 IBD cases and controls, including data from 15 different genome-wide association scans (GWAS) for ulcerative colitis and Crohn’s disease, the International IBD Genetics Consortium (IIBDGC) identified 71 new causative regions, increasing the total number of independent IBD risk loci to 163: 110 associated with both diseases, 30 classified as Crohn’s disease-specific, and 23 as ulcerative colitis-specific. The notable overlap of genetic loci suggests that Crohn’s disease and ulcerative colitis share many biological mechanisms: 43 of the 53 disease specific loci have the same direction of effect in both diseases, suggesting concordance for many of the biological mechanisms implicated in both diseases. Insight into biological differences is supported by the observation that two risk loci for Crohn’s disease, *NOD2* and *PTPN22*, are protective for ulcerative colitis ^17– 19^. These strategies have identified several important signaling pathways that have consistently been associated with susceptibility to IBD. Figure 2 depicts genetic loci associated with IBD, grouped by disease specificity and involved pathways ^19^.

Some of these pathways highlight the interaction between the host, the microbiome, and their products. Genetic analysis has highlighted the importance of autophagy in immune responses in IBD. Autophagy, involved in intracellular homeostasis, facilitates the degradation and recycling of cytosolic contents and organelles, and also helps resist microbial infection by removing intracellular microbes ^20^. In Crohn’s disease, several genes ( *ATG16L1, IRGM*, and *LRRK*) regulate the autophagy pathway, including *NOD2*, further supporting the theory of defective microbial degradation in some patients with Crohn’s disease ^21– 26^.

Genetic loci also affect innate and adaptive immunity and epithelial function. Specifically, *NOD2* modulates innate and adaptive immune responses ^14, 15^. Further, adaptive immune genes that regulate the interleukin (IL)-17 and IL-23 receptor pathways are implicated in IBD risk, including genes associated with risk for ulcerative colitis and Crohn’s disease (e.g., *IL23R, IL12B, STAT3, JAK2*, and *TYK2*) and those implicated in Crohn’s disease only (e.g., *IL-27, TNFSF15*). A number of genes associated with epithelial barrier function are also specifically associated with only ulcerative colitis and not with Crohn’s disease (e.g., *OCTN2, ECM1, CDH1, HNF4A, LAMB1*, and *GNA12*) ^13, 27, 28, 30, 31^. Genes that control Paneth cell biology and the endoplasmic reticulum (ER) stress/unfolded protein response are also associated with Crohn’s disease (e.g., *Xbp-1; Nod2*) ^32^.

Different compositions of gut microbiota affect epigenetic regulation of genes through microbial products such as SCFAs ^33^. Butyrate, one type of SCFA, influences epigenetic methylation of SCFA receptors, especially the promoter region of the free fatty acid receptor 3 (FFAR3) with consequent effects on gene expression and function ^34^. We will further discuss SCFAs and their specific pathways later in this review.

Despite the above-mentioned advances, no genetic associations can be confirmed in 77% of Crohn’s disease patients and in up to 84% of ulcerative colitis patients ^30^. Alterations in 163 distinct single genes confer only a modest effect in and of themselves, suggesting that an aggregate effect at several loci may be responsible for the IBD phenotype ^35^. For instance, as many as 20 to 30% of patients with Crohn’s disease may have a variant *NOD2*, though the penetrance is not more than 5% of homozygous and roughly 0.5% in heterozygous persons ^31^. This indicates that disease-related allelic variants of the gene may be present in a large number of persons who do not have Crohn’s disease.

While the increasing number of susceptibility gene loci described in IBD reflects their importance, the loci discovered so far account for only 20–25% of IBD heritability ^30^. Further, the remarkable rise of the incidence of IBD over the past few decades cannot be sufficiently explained by only genetic risk or increased diagnosis and accessibility of care ^2^, which has opened the doors for immunological, environmental and particularly microbial-based research in this field.

## Microbiome

A microbial etiology for IBD has long been hypothesized, starting with descriptions of potential infectious agents associated with ulcerative colitis in the 19 ^th^ century and Crohn’s disease in the early 20 ^th^ century ^36, 37^. In the 1920s, Rettger *et al.* studied the effects of *Bacillus acidophilus* on IBD, while in the 1940s Kirsner evaluated the possible correlation between streptococci and ulcerative colitis ^38– 40^. In the late 1990s, the association between fecal microbiota and Crohn’s disease was apparent when recurrent inflammation was observed after the fecal stream was reestablished in post-operative Crohn’s disease patients ^41, 42^. Despite these associations, no specific microbe(s) were identified to be the cause of IBD.

**Figure 3.**
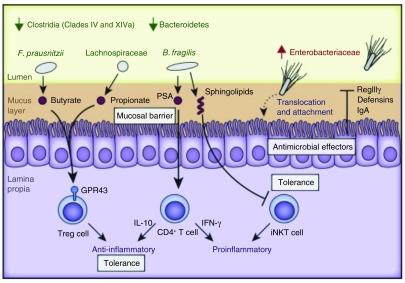
Dysbiosis and gut inflammation. The schematic depicts consistent observations of changes in microbial composition underlying dysbiosis and associated pathways modulating gut inflammation. The lumen (yellow), mucus layer (brown), epithelium (purple brush-border-containing cells), and lamina propria (bottom purple section) are indicated. Multiple mechanisms depicted include regulation by T cells, short chain fatty acids (SCFAs), sphingolipids and antimicrobial factors. Adapted from 85.

With recent advances in bioinformatics and culture-independent methods used for bacterial identification, there has been a resurgence of interest in the 21 ^st^ century in studying the phylogeny and function of the gut microbiome in IBD. One popular proposed mechanism is the development of dysbiosis, which is defined as an imbalance between protective and harmful intestinal bacteria causing disease. Figure 3 depicts proposed microbial composition changes underlying dysbiosis and associated pathways modulating gut inflammation, including regulation by T cells, SCFAs, sphingolipids and antimicrobial factors as reviewed recently by Huttenhower *et al.* ^43^.

The diversity of the intestinal microbiome is 30–50% lower in IBD subjects than in controls. In the past year, a study investigating twin pairs discordant for IBD revealed a reduction in microbial diversity in the healthy sibling, mirroring the changes in the ulcerative colitis-affected twin ^44^. Furthermore, individuals who are steroid-responsive have a more diverse microbiota when compared to non-responders (Shannon index 338 ± 62 *versus* 142 ± 49; *P* = 0.013) ^45^.

There is also evidence that upregulation and downregulation of the abundance of certain bacterial species correlates with disease activity. Recent studies have demonstrated a significant reduction of *Faecalibacterium prausnitzii* and *Roseburia hominis* in active ulcerative colitis patients *versus* control subjects. Moreover, a significant inverse correlation between disease activity and the abundance of *R. hominis* and *F. prausnitzii* is present even in quiescent ulcerative colitis ^46, 47^. *F. prausnitzii* is widely regarded as one of the main fecal bacterial groups involved in colonic saccharolytic fermentation which produces SCFAs, in particular, butyrate ^48^.

**Figure 4.**
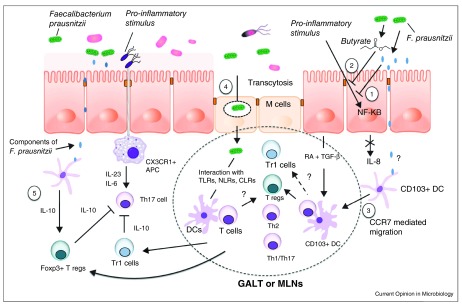
Proposed anti-inflammatory mechanisms of *F. prausnitzii*. **1.** The supernatant of *F. prausnitzii* blocks NF-κB activation induced by inflammation. **2.** Butyrate produced by *F. prausnitzii* inhibits NF-κB activation in the mucosa. **3.** *F. prausnitzii* may interact with CD103+ dendritic cells (DCs) in the lamina propria and stimulate their migration to mesenteric lymph nodes (MLN) and the induction of Tregs. **4.** M cell transcytosis of *F. prausnitzii* in organized lymphoid structures may induce Tregs. **5.** *F. prausnitzii* may induce IL-10 in antigen-presenting cells to enhance the suppressive activity of Foxp3+ Tregs and block Th17 cells. Adapted from 93.

Further validation of the protective function of some microbial genera of the microbiome in acute and chronic colitis was confirmed by the improvement of inflammatory markers after intragastric administration of *F. prausnitzii*. In their mouse studies, Sokol *et al.* reported the protective effect of *F. prausnitzii* in a trinitrobenzene sulfonic acid (TNBS)-induced acute colitis model and, more recently, in a model of dinitrobenzene sulfonic acid (DNBS)-induced chronic colitis, in which a reduction of inflammatory markers, such as myeloperoxidase (MPO) and pro-inflammatory colonic cytokines (IL-6, IL-9, TNF-α, IFN-α), was reported, indicating a decreased severity of inflammation associated with an alteration of the microbiome ^49, 50^. The findings were particularly notable in that further analysis indicated that butyrate was not implicated in this protective effect, presumably due to the limitations of the TNBS colitis model employed, but nonetheless suggesting other protective mechanisms are present. Figure 4 briefly summarizes the numerous proposed anti-inflammatory mechanisms mediated by *F. prausnitzii*, either by its metabolites or by direct contact with the mucosa ^47^. These pathways, ranging from production of anti-inflammatory matrix components to SCFAs to regulation of the immune system, to activation of the inflammatory cascade and the enteric nervous system, represent the complexities inherent in elucidating the biological mechanisms relating the microbiome to IBD pathogenesis ^47, 51– 53^.

A multicenter cohort study that enrolled treatment-naïve and newly diagnosed patients with Crohn’s disease reported increased abundance of Enterobacteriaceae, Pasteurellacaea, Veillonellaceae, and Fusobacteriaceae and decreased abundance of Erysipelotrichales, Bacteroidales, and Clostridiales in ileal and rectal biopsies ^54^. The complexities underlying the interpretation of such simple microbial associations through their production of SCFA are evident in conflicting observations of increased Enterobacteriaceae and Fusobacteriacea in Crohn’s disease, both of which are implicated as the main SCFA-producing bacterial groups ^48^.

The importance of the luminal contents and the microbiome in the foregut is also illustrated in a recent study by Said *et al.* reporting that dysbiosis in the oral cavity is associated with inflammatory responses in IBD patients. The salivary microbiome of patients with IBD had higher proportions of *Prevotella, Bacteroidetes,* and *Veillonella* and lower proportions of *Streptococcus*, *Neisseria*, *Haemophilus, Proteobacteria,* and *Gemella* ^55^. Although the study reported changes in several bacterial groups that may have obscured the effect of a single group, *Bacteroidetes* is one of the major groups producing SCFAs ^48^, which are thought to protect against inflammation.

Dysbiosis is also suggested by the observations of increased prevalence of bacteria that may be implicated in the pathogenesis of IBD. For instance, in ulcerative colitis patients, the population of sulfite-reducing bacteria such as *Desulfovibrio* is increased, whereas low amounts of thiosulfate sulfur transferase (TST), an enzyme responsible for hydrogen sulfide (H _2_S) detoxification, are present ^56– 58^. The consequent increased intestinal H _2_S content can impair DNA repair and inhibit SCFA oxidation and its protective properties ^59, 60^, further implicating microbial products such as SCFAs as key mediators.

Some aspects of the specific changes of microbial composition triggering IBD, however, continue to be elusive: for instance, it is yet to be established whether the gut microbiome is stable or continuously changing during the course of the disease. Furthermore, the impact of diet, standard medical therapy, and other environmental factors on the gut microbiome is not well understood. Most importantly, it is as yet undetermined if microbial imbalance is a cause or a consequence of IBD development.

Although contemporary research has focused mostly on descriptive study of the compositional changes in gut microbiota in IBD, studies of the functional impact of microbial communities in IBD will be necessary to gain further insight into disease pathogenesis. On the basis of metagenomic and metaproteomic studies, only 2% of genera changes in stool and intestinal biopsy specimens may have a much larger functional impact, affecting up to 12% of total metabolic pathways in active IBD patients compared to controls ^61^.

## Integrating the microbiome and the genome

Isolated research on the genetic and microbial factors affecting IBD manifestations and pathogenesis over the past decades has provided valuable insights and strong associative relationships between IBD, genetics, and dysbiosis, but has been unable to provide mechanistic explanations for these associations. There have been limited studies of the co-association of complex host genetic factors with microbial composition and metabolism in IBD patients or other populations. IBD-associated genetic variants associated with alterations in the intestinal microbiome, particularly in individuals carrying polymorphisms in *NOD2* and *FUT2*, have been reported ^62, 63^. ** The mechanisms of host genome-microbiome disease pathways are largely unknown.

There is mounting evidence that genetic loci across the human genome are instrumental in shaping the gut microbiome ^64, 65^. Knights *et al.*, in a systematic analysis of the effect of 154 IBD-associated polymorphisms on microbial composition in three cohorts of patients with IBD (152 to 162 patients in each cohort) using multivariate linear models, reported that 49/154 IBD-associated genes significantly and concordantly affected microbial taxa in at least two of the cohorts, implicating the innate immune response, the inflammatory response, and the JAK-STAT cascade ^64^. In a separate analysis, the *NOD2* risk allele count also influenced the overall microbial composition and abundance of Enterobacteriaceae. These data not only support an intricate link between host genetics and microbial dysbiosis in IBD, but also illustrate the ability to uncover novel associations from paired genome-microbiome data, opening the possibility that an unexpected number of genetic factors act directly on microbial composition, modulating immune pathways and metabolic phenotypes in host physiology and disease. Further studies are necessary to understand if these variants contribute to disease phenotype through their direct influence on microbiome selection, which in turn can affect disease pathophysiology either through elaboration of metabolic products or through direct mucosal interaction. These studies may involve investigating whether genetic polymorphisms concordantly affect microbial composition in healthy individuals in addition to those with IBD.

## Biological reductionism

The systems-based studies of genome and microbiome in the pathogenesis of IBD have only yielded associations without a causal mechanism. Traditional reductionist experimentation is necessary to validate associations in system-based approaches. With the advent of new statistical methods, computing and technological advances termed “systems biology”, this approach has become dominant. Since complex models with emergent properties are arguably difficult to explain with a reductionist approach, the systems approach looks broadly for correlations in comprehensive data sets, building models based on these correlations. The statistical approaches used to “mine” systems-based data sets are tools from which hypotheses can be developed. These hypotheses should then be tested in specific (and often reductionist) experiments. Thus, experimental verification of the systems-based approaches will be important to establish if the statistical approaches employed in data analysis are robust. It is therefore the marriage of systems-based approaches with traditional reductionist experimentation that will be needed to advance the field.

## SCFAs: pathway to IBD

Few studies have addressed the gap between intestinal microbes and inflammatory biological pathways in the understanding of IBD pathogenesis in a human host. Dysbioses in IBD are not simply structural changes in the gut microbiota, but are instead associated with major impairments of many fundamental microbial metabolic functions with potential impact on the host. Profound disturbances have been reported in the metabolic pathways associated with gut microbiota in IBD, including major shifts in oxidative stress pathways, decreased amino acid biosynthesis, increased mucin degradation, and decreased SCFA production ^61^.

A promising route to further understanding the pathogenesis of IBD involves the investigation of the interactions of gut microbiota with the host, particularly through the bacterial fermentation products *N*-butyrate and other SCFAs. Not only do SCFAs provide essential nutrition for colonocytes, but they are also sensed by enteroendocrine and enterochromaffin cells in addition to possessing anti-inflammatory activity *in vitro* and *in vivo* ^48, 66^.

SCFAs have been studied for decades for their effects on IBD. Although early clinical trials reported beneficial effects of SCFA enemas in ulcerative colitis patient subpopulations (e.g., distal ulcerative colitis, mild-to-moderate distal ulcerative colitis ^67, 68^), several large randomized studies reported no significant effects of exogenous SCFA treatment of ulcerative colitis patients ^69, 70^. These early trials were confounded by the now known epigenetic regulation of multiple host factors by SCFAs. Other limitations include the unknown utility of a transient rise of SCFA concentration in the distal gut achievable through enemas compared to sustained foregut elevations made possible by specific microbiome compositions of specific gut segments.

SCFAs, fermented from dietary fiber resistant to mammalian digestion, are actively produced by anaerobic microbiota in the intestine and colon. The concentration of SCFA in hindgut lumen can reach 100 mM, which provides sufficient driving force for absorption by or transport into colonocytes ^71^. SCFAs activate specific G-protein-coupled receptors (GPCRs), in particular GPR43 (FFA2), expressed by leukocytes, adipocytes and enterochromaffin (EC) cells, myenteric nerves, and GPR41 (FFA3), expressed by adipose tissue, spleen, bone marrow, lymph nodes, enteroendocrine cells, and peripheral blood mononuclear cells. GPR signaling can regulate cell activation, proliferation, and differentiation through the release of hormones or other bioactive molecules, or possibly through direct effects on enteric nerves ^48^.

Another mechanism of SCFA action is inhibition of histone deacetylase (HDAC) activity, with subsequent modification of gene expression in human cells ^48, 50, 72^. Because HDAC inhibition increases the acetylation of histone and other proteins, it can impact multiple genes and proteins. SCFAs also regulate cell metabolism through the Krebs cycle intermediates and mechanistic target of rapamycin (mTOR) activation regulating T cells ^72^.

Our laboratory has pursued experimental studies aimed at decoding how chemosensing of luminal microbial products, including SCFAs, can generate host responses. We have shown that the duodenum possesses specialized chemosensing functions that alert the distal gut to proximal conditions. The presence of SCFA in the proximal gut lumen activates mucosal defense mechanisms, including increased mucosal blood flow and mucus, bicarbonate secretion, and release of gut hormones ^73– 77^. One notable mechanism implicates the expression of SCFA receptors in luminal-facing projections of rat duodenal EC cells and enteroendocrine L-cells. FFA3 colocalises with glucagon-like peptide (GLP)-1 in enteroendocrine cells, whereas FFA2 colocalises with 5-hydroxytryptamine (serotonin; 5-HT) in EC cells. Activation of FFA2 receptor expressed on EC cells releases 5-HT and acetylcholine (ACh). These activate 5-HT _4_ and muscarinic receptors respectively, which are expressed on enteric nerves, afferent nerves, and epithelial cells. Activation of duodenal epithelial cells by these signals increases the rate of HCO _3_^−^ secretion.

The contribution of 5-HT and ACh was confirmed subsequently when a synthetic selective FFA2 agonist dose-dependently increased HCO _3_^−^ secretion, but was inhibited by atropine and a 5-HT _4_ antagonist. Similarly, SCFAs are thought to activate FFA2 receptors expressed on L cells, releasing GLP-2, which activates GLP-2 receptors expressed in myenteric neurons, enhancing HCO _3_^-^ secretion, inhibited by GLP-2 receptor antagonists but enhanced by dipeptidyl peptidase (DPP) IV inhibition. These novel pathways have an inherent ability to locally regulate hormone release, implying that they are important in mucosal homeostasis ^74^. Dysregulation of these pathways may contribute to intestinal inflammation through possible mechanisms detailed below, highlighting 5-HT and GLP-2 mediated effector pathways.

Serotonin (5-HT) mediates many GI functions, including secretion and peristalsis, presumably through its activation of the five known gut-expressed 5-HT receptors out of the seven 5-HT receptors so far described ^78^. Agonists and antagonists to 5-HT _3_ and 5-HT _4_ receptors are particularly well studied, with many drugs in clinical use, with utility in the management of diarrhea, constipation, and gut associated pain syndromes ^78^. The contribution of 5-HT and its most recently discovered 5-HT _7_ receptors to intestinal homeostasis and inflammation is less well understood. Initial studies reported alterations in 5-HT signaling in IBD; differences in EC cell and 5-HT content have been reported with ulcerative colitis and Crohn’s disease ^79– 85^. 5-HT released from EC cells can act on proximal gut 5-HT _7_ receptors expressed by smooth muscle cells, enteric neurons, enterocytes, and immune cells. Activation of 5-HT _7_ receptors can influence muscle tone and enteric neuron excitation, inhibit serotonin transporter (SERT) activity, and modulate inflammation through dendritic cells (DCs) in the lamina propria, which are highly involved in host immune pathways.

Guseva *et al.* investigated the enhanced expression and distribution of 5-HT _7_R in the intestinal tissue of IBD patients, based on an experimental model of dextran sulfate sodium (DSS)-induced colitis in which 5-HT _7_R expression was upregulated on CD11c/CD86 double-positive dendritic cells obtained from cecal and rectal tissue samples. The authors reported a similarly high expression of 5-HT _7_R in analogous dendritic cells obtained from large intestinal tissue samples of patients diagnosed with Crohn’s disease. Pharmacological blockade or genetic ablation of 5-HT _7_R increased the severity of acute and chronic DSS-induced colitis, whereas receptor stimulation was anti-inflammatory. These experiments supported the hypothesis that 5-HT _7_R expressed on CD11c/CD86-positive myeloid cells is an important component of intestinal inflammatory pathways ^86^. Contrary to these results, Kim *et al.* in 2013 reported that pharmacologic blockade or genetic ablation of 5-HT _7_R actually alleviated intestinal inflammation in two separate chemical models of colitis (DSS and DNBS), which was confirmed histopathologically and also associated with decreased concentrations of pro-inflammatory markers, including myeloperoxidase (MPO) and cytokines IL-1β, IL-6, and TNF-α. Mice that received hematopoietic stem cells from 5-HT _7_ receptor-deficient donors exhibited decreased histopathological damage and disease activity ^87^. These apparently discordant effects may be due to differences in dosing of the 5-HT _7_R antagonist despite substantial differences in experimental design and housing condition of the animals. In separate experiments by Kim *et al.* in which a lower dose (20mg/kg) of the antagonist was used, no significant differences were detected compared to control, though a higher dose (80mg/kg) significantly decreased colitis severity and inflammatory markers. Kim further acknowledged that the antagonist dosages were higher and dosing periods longer in his studies than those used by Guseva *et al.* ^85^. Thus, 5-HT _7_R expressed on CD11c/CD86-positive myeloid cells, which modulate the severity of intestinal inflammation in experimental models of acute and chronic colitis, may serve as a potential therapeutic target for the treatment of inflammatory disorders such as Crohn’s disease.

The other hormone released through the action of SCFA, GLP-2, promotes mucosal growth, decreases barrier permeability and reduces inflammation in the intestine ^88^. After recently being approved for treatment of short bowel syndrome, there has been a surge in interest in the non-metabolizable GLP-2R agonist teduglutide due to its multiple beneficial effects, including effects on glucose homeostasis ^88^, and on intestinal inflammation in Crohn’s disease. Pediatric patients with acute ileal Crohn’s disease have lower postprandial GLP-2 release and higher intestinal permeability, which both reverse as the disease improves, suggesting a crucial link between the inflammatory state and GLP-2 meal-stimulated release ^89^. In another study, GLP-2 treatment was associated with a significantly reduced neutrophil infiltration and microscopic colitis scores in the TNBS model of colitis in mice. They also reported that GLP-2 contributed to protecting the enteric nervous system under basal conditions or inflamed states ^90^. GLP-2 is not only trophic for the intestine but also has other salutatory effects. The GLP-2R, expressed by pericryptal myofibroblasts, releases growth factors in response to GLP-2R activation ^91^. GLP-2 decreases gut inflammation by downregulation of Th1 cytokines cells *via* an IL10-independent pathway, altering the mucosal response of inflamed intestinal epithelial cells and macrophages ^92^. GLP-2 also may activate enteric nerves, since the GLP-2R is localized to myenteric neurons in addition to the myofibroblasts, but not to the intestinal epithelium ^93^.

**Figure 5.**
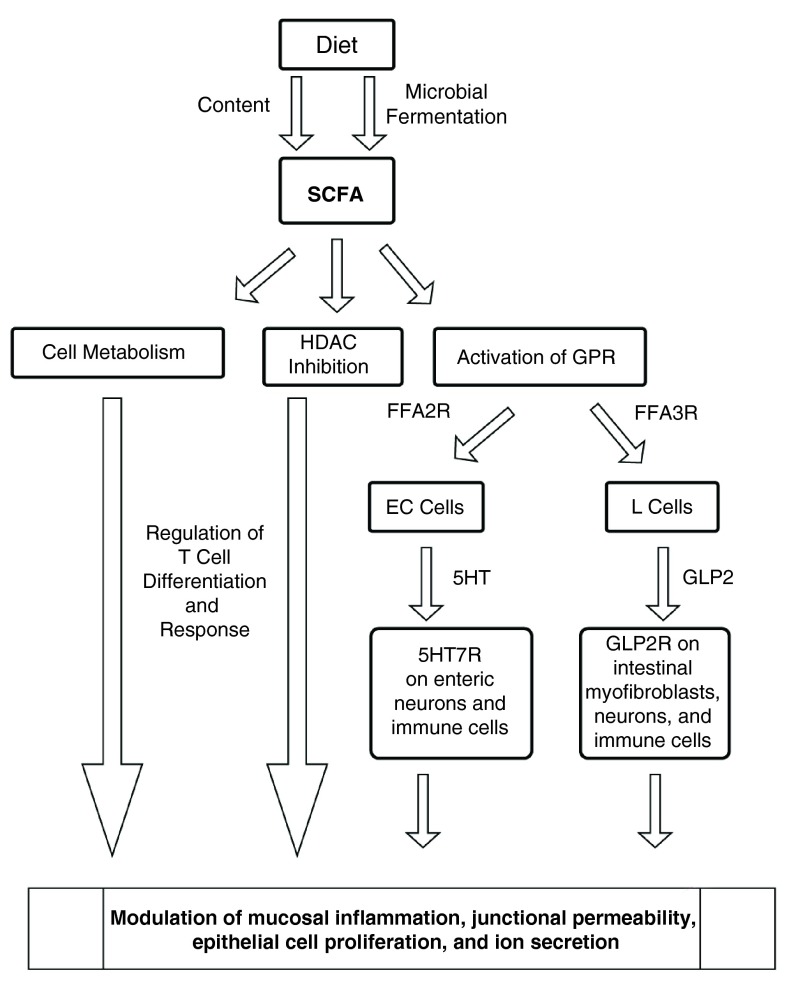
Short chain fatty acids (SCFAs) regulate gut inflammation. SCFAs are present in the diet and actively produced by gut microbiota as fermentation products of dietary materials. SCFAs exert their effects directly on epithelial cells, antigen-presenting cells, and T cells. Multiple mechanisms depicted above include metabolic regulation, HDAC inhibition, and GPR activation by SCFAs releasing mediators including 5-HT and GLP-2 hypothesized to modulate gut inflammation.

The contribution of specific gut microbiota is evident in studies that demonstrate their influence on 5-HT biosynthesis and on the increase in endogenous GLP-2 production—both of which are implicated in modulation of gut inflammation—although the signaling pathways are yet to be fully understood ^94– 96^. SCFAs may represent the biological mediators of these findings as well. SCFAs that promoted Tph1 (tryptophan hydroxylase 1) transcription in BON cells (human EC cell model) were the key link between gut microbiota regulating enteric 5-HT production and homeostasis in a recent study ^94^. Figure 5 outlines the proposed mechanisms through which SCFAs regulate gut inflammation.

## Other pathways

There are many other pathways being investigated using the reductionist approach to help integrate genetic, microbial, and biochemical pathways. In a breakthrough study of IBD- protective single nucleotide polymorphisms (SNPs) in the *MAP3K8* gene, Roulis *et al.* reported that *MAP3K8* encodes tumor progression locus-2 (Tpl2) kinase in intestinal myofibroblasts, in addition to promoting arachidonic acid metabolism and COX-2/PGE-2 activation, which are important in the compensatory proliferative response of the intestinal epithelium to injury ^97^.

Mice with complete knockout of Tpl2, and conditional knockout of Tpl2 targeted to intestinal myofibroblasts, were highly susceptible to DSS-induced colitis, with greater tissue damage when compared to wild-type mice despite similar DSS-induced levels of inflammation. Tpl2 expression was downregulated in intestinal myofibroblasts isolated from the inflamed ileum of nine patients with IBD.

## Conclusions

Currently, the data acquisition rate in traditional IBD research has been far outpaced by the massive data generated by bioinformatics. Despite the considerable ongoing efforts of investigators across the globe, reductionist studies are still needed to help explain the basic causal mechanisms that underlie the exponentially increasing number of correlations detected through systems-based approaches in IBD. Further integration of the study of host genetics and the gut microbiome in the setting of clinical metadata has already reduced the number of confounding variables and has elicited new associations. Efforts to integrate complicated genetics and the gut microbiome in further reductionist experiments to determine causal associations may be best served by an emphasis on bacterial metabolic products, such as SCFAs, as the logical mediators of the interactions between genetic variables and the microbiota as well as key molecules of the biological etiopathogenic pathway to gut inflammation. Further reductionist studies exploring the interactions between diet, microbiome, SCFAs and serotonin can help guide our understanding of the pathogenesis of colitis.
